# Recombinant Laminins Drive the Differentiation and Self-Organization of hESC-Derived Hepatocytes

**DOI:** 10.1016/j.stemcr.2015.10.016

**Published:** 2015-11-25

**Authors:** Kate Cameron, Rosanne Tan, Wolfgang Schmidt-Heck, Gisela Campos, Marcus J. Lyall, Yu Wang, Baltasar Lucendo-Villarin, Dagmara Szkolnicka, Nicola Bates, Susan J. Kimber, Jan G. Hengstler, Patricio Godoy, Stuart J. Forbes, David C. Hay

**Affiliations:** 1 MRC Centre for Regenerative Medicine, University of Edinburgh, Edinburgh EH16 4UU, UK; 2 Leibniz Institute for Natural Product Research and Infection Biology eV-Hans-Knöll Institute, 07743 Jena, Germany; 3 IfADo-Leibniz Research Centre for Working Environment and Human Factors at the Technical University Dortmund, Ardeystraße 67, 44139 Dortmund, Germany; 4 Faculty of Life Sciences, Michael Smith Building, Oxford Road, Manchester M13 9PT, UK

## Abstract

Stem cell-derived somatic cells represent an unlimited resource for basic and translational science. Although promising, there are significant hurdles that must be overcome. Our focus is on the generation of the major cell type of the human liver, the hepatocyte. Current protocols produce variable populations of hepatocytes that are the product of using undefined components in the differentiation process. This serves as a significant barrier to scale-up and application. To tackle this issue, we designed a defined differentiation process using recombinant laminin substrates to provide instruction. We demonstrate efficient hepatocyte specification, cell organization, and significant improvements in cell function and phenotype. This is driven in part by the suppression of unfavorable gene regulatory networks that control cell proliferation and migration, pluripotent stem cell self-renewal, and fibroblast and colon specification. We believe that this represents a significant advance, moving stem cell-based hepatocytes closer toward biomedical application.

## Highlights

• We designed a hepatocyte differentiation process under defined conditions
• The hepatocyte phenotype is improved and stabilized on two laminin isoforms
• Laminin-supported differentiation suppresses inappropriate gene networks
• Laminin surfaces deliver hepatocytes with greater similarity to adult hepatocytes

## Teaser

In this article, Hay and colleagues demonstrate that hepatocyte production is possible under defined conditions with GMP-grade hESC lines. The use of recombinant laminins significantly improved the stem cell-derived hepatocyte phenotype, which was closer in nature to primary adult hepatocytes.

## Introduction

Significant advances in cell-based therapies, particularly in the liver, represent promising alternatives to whole-organ transplantation. Cell transplantation has several benefits over organ transplantation, including the use of one organ for several patients, and the procedure itself is generally less invasive. Although significant progress has been made, immune system clearance and cell-based therapy reliance upon organ donations are significant limitations. As a result, obtaining a potentially unlimited supply of somatic cells from defined genetics for cell-based therapy is an intense area of study.

Pluripotent stem cells are particularly promising cell types, possessing the ability to self-replicate and differentiate into all cell types in the body, including hepatocytes (Sun et al., 2013). This promises in theory an “off-the-shelf” alternative to donated tissue. Differentiation procedures have advanced over the last decade, and efficient protocols to generate stem cell-derived hepatocyte-like cells (HLCs) from either human embryonic stem cells (hESCs) or induced pluripotent stem cells (iPSCs) now exist (Lavon et al., 2004, Hay et al., 2007, Hay et al., 2008, Hay et al., 2011, Cai et al., 2007, Duan et al., 2007, Basma et al., 2009, Sullivan et al., 2010, Touboul et al., 2010, Si-Tayeb et al., 2010, Rashid et al., 2010, Payne et al., 2011, Zhou et al., 2012, Zhou et al., 2014, Medine et al., 2013, Takayama et al., 2013a, Szkolnicka et al., 2014a). These procedures utilize growth factors, mimicking each stages of embryonic development, and can deliver homogenous populations of HLCs. Although these prototype systems have provided confidence in pluripotent stem cell technologies, their amenability to defined scale-up with clinical-grade hESC lines has not been achieved.

HLCs derived from pluripotent stem cells have shown significant promise in vitro, accurately modeling human drug exposure (Medine et al., 2013, Szkolnicka et al., 2014b, Holmgren et al., 2014, Ware et al., 2015). HLCs have also been employed to study the hepatitis C virus life cycle (Wu et al., 2012, Roelandt et al., 2012, Zhou et al., 2014, Carpentier et al., 2014) and, more recently, the malaria parasite (Ng et al., 2015). HLCs have also been derived from patients with monogenic metabolic liver diseases and have been shown to recapitulate features of α1-antitrypsin deficiency, familial hypercholesterolemia, and glycogen storage disease (Rashid et al., 2010). The ability to generate population-specific HLCs that accurately represent adult liver tissue has significant implications in the drug development process and in stratifying patient healthcare.

Despite these advances, HLCs derived from pluripotent stem cells still display an immature phenotype (Godoy et al., 2015, Forbes et al., 2015). This phenotypic immaturity has contributed to the limited the use of stem cell-derived hepatocytes for clinical application (Schwartz et al., 2014). Efforts to address this have utilized natural and synthetic culture substrates to improve mature hepatocyte function and enhance stability (Takayama et al., 2013b, Jitraruch et al., 2014, Villarin et al., 2015). Other studies have used small molecules to replace growth factors to drive down the cost of the method (Hay et al., 2007, Hay et al., 2008, Siller et al., 2015); however, those published methods both still rely upon the use of undefined components, highlighting the need to develop defined systems.

A critical component of in vitro maintenance and differentiation systems is extracellular matrix (ECM) support. The ECM elicits profound effects on cell behavior. In the human stem cell self-renewal and differentiation fields, the mouse sarcoma-derived Matrigel represents the most extensively used ECM. Although Matrigel has undoubtedly been enabling for the stem cell field, its relatively undefined nature and significant batch-to-batch variability results in difficulties to generate reliable, reproducible cultures of HLCs. Moreover, if HLCs are to be utilized clinically, then this process must meet good manufacturing process (GMP) guidelines. To comply with this, products containing animal derivatives are strictly controlled. Therefore, to overcome variability, and in an effort to define our differentiation process, we employed two recombinant full-length human laminins and compared those with Matrigel in three hESC lines, two of which are available at GMP-grade.

The laminins used in our system were selected because of their importance in the developing embryo (Domogatskaya et al., 2012, Miner et al., 2004), the regenerating liver (Martinez-Hernandez and Amenta, 1995, Carlsson et al., 1981), and the liver stem cell niche (Tanimizu et al., 2012, Lorenzini et al., 2010). Additionally, the laminins employed in this study are known to support the clonal expansion of hESCs (Rodin et al., 2010) and drive endodermal differentiation (Taylor-Weiner et al., 2013) and liver stem cell differentiation (Takayama et al., 2013a), making them ideal candidate substrates for our purposes. Although endoderm and liver stem cell differentiation has been achieved, neither of the laminin matrices employed, in isolation or mixed format, have been shown to support hepatocyte differentiation from research- and GMP-grade hESC lines.

Utilizing pure laminin 521 (L521) and the blend of laminins 521 and 111 as substrates for hESC-derived differentiation, we demonstrate efficient hepatocyte specification and significant improvements in cell function and phenotype. Furthermore, stem cell-derived hepatocytes derived on laminin surfaces formed organized structures, and their gene regulatory networks were closer to that of freshly isolated human hepatocytes. We believe that these studies represent a significant advance toward the large-scale production of clinical-grade and quality-assured human hepatocytes from GMP hESCs.

## Results

### Differentiation of Pluripotent Stem Cells Toward Hepatocytes

**Figure 1:**
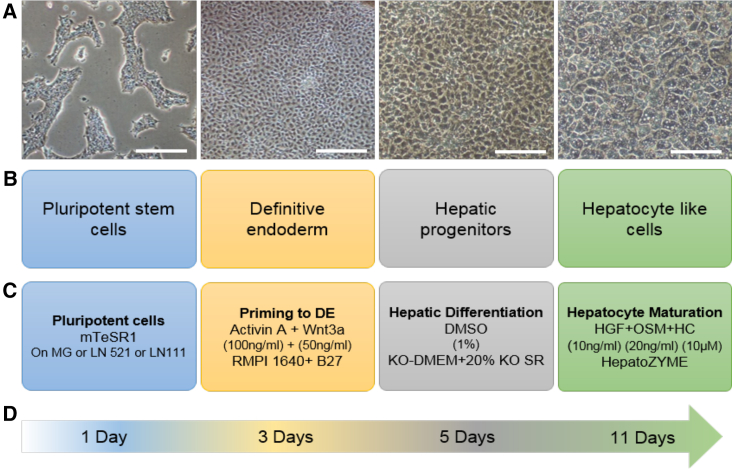
Differentiation Protocol (A) Phase contrast images of representative fields of view at each key stage during differentiation (magnification, ×10). Scale bars, 100 μm. (B) Cell types present at differentiation stage. (C) Growth factors or molecules and media used at each stage. MG, Matrigel; DE, definitive endoderm; SR, serum replacement; HC, hydrocortisone. (D) Timescale of the process.

**Figure 2:**
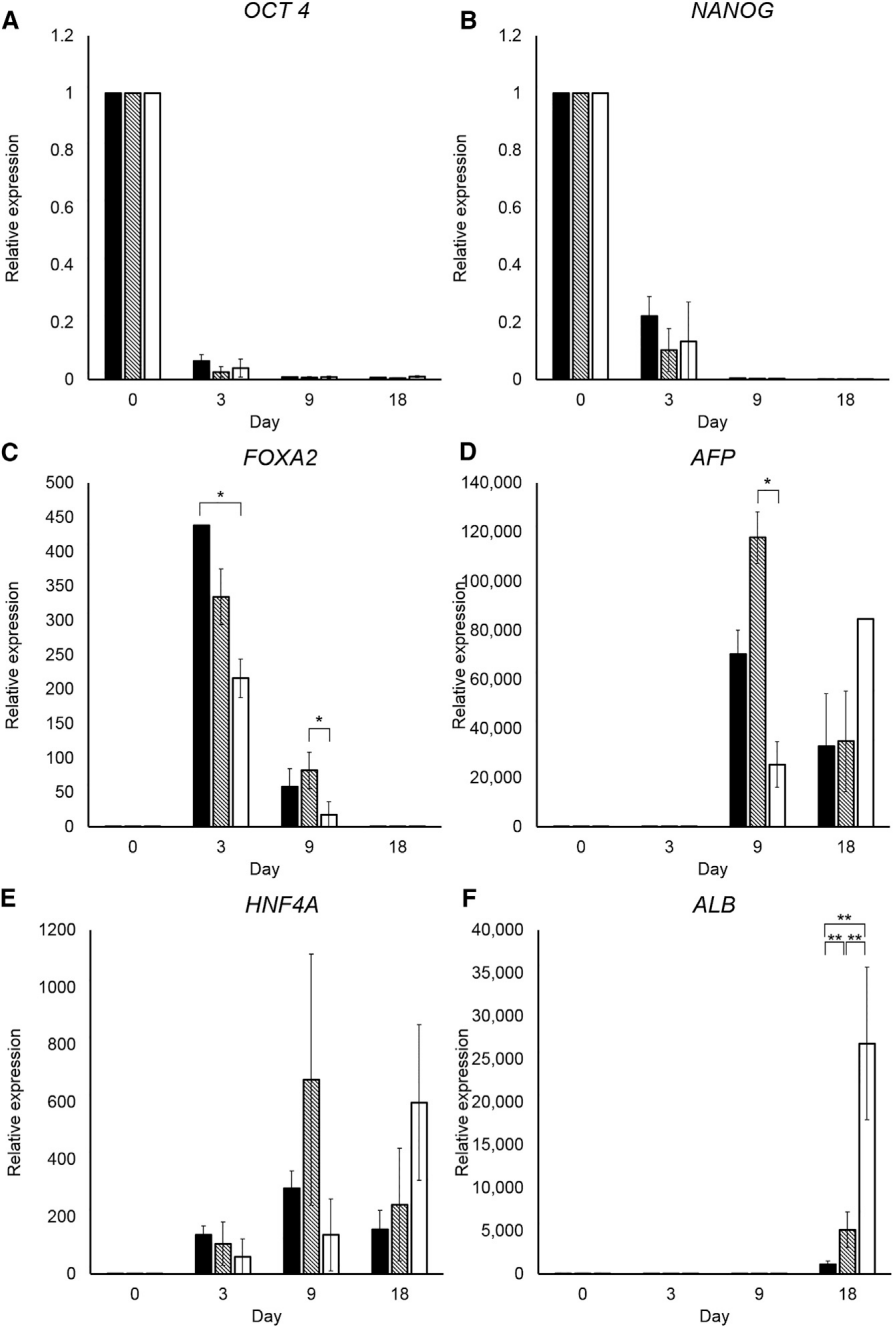
Gene Expression during Differentiation (A–F) Gene expression of the pluripotency-associated markers (A) *OCT4* and (B) *NANOG*, the endoderm/hepatoblast markers (C) *FOXA2* and (D) α fetoprotein (*AFP*), (E) hepatocyte nuclear factor-4-α (*HNF4A*), and (F) albumin (*ALB*) was analyzed on days 0, 3, 9, and 18, normalized to the housekeeping gene *GAPDH*, and expressed relative to hESCs. The results shown represent three biological replicates, and error bars represent SD. ^∗^p < 0.05, ^∗∗^p < 0.01; one-way ANOVA with Tukey post hoc test.

hESCs were replated onto Matrigel and wells coated with pure laminin 521 and the laminin 521:111 mix (1:3 ratio, hereafter referred to as laminin 111 mix [L111]). hESCs were differentiated using our standard protocol, outlined in Figure 1, and analyzed at key time points. hESCs on all three matrices adhered, proliferated, and differentiated into HLCs. Twenty-four hours after replating, hESCs were examined for characteristic colony morphology and stem cell-associated marker expression (Figure S1). hESCs displayed the appropriate embryonic stem cell morphology, with subtle differences detected on each matrix (Figure S1A). Importantly, the majority of hESCs expressed the stem cell markers Oct4 and Nanog when attached to Matrigel (88.3% ± 4.8% and 86.7% ± 5.0%, respectively), laminin 521 (93.2% ± 5.9% and 88.7% ± 5.4%, respectively), and laminin 111 mix (93.8% ± 2.4% and 90.4% ± 3.5%, respectively) (Figure S1B; Table S1). These results were corroborated by qPCR (Figures 2A and 2B).

**Figure 3:**
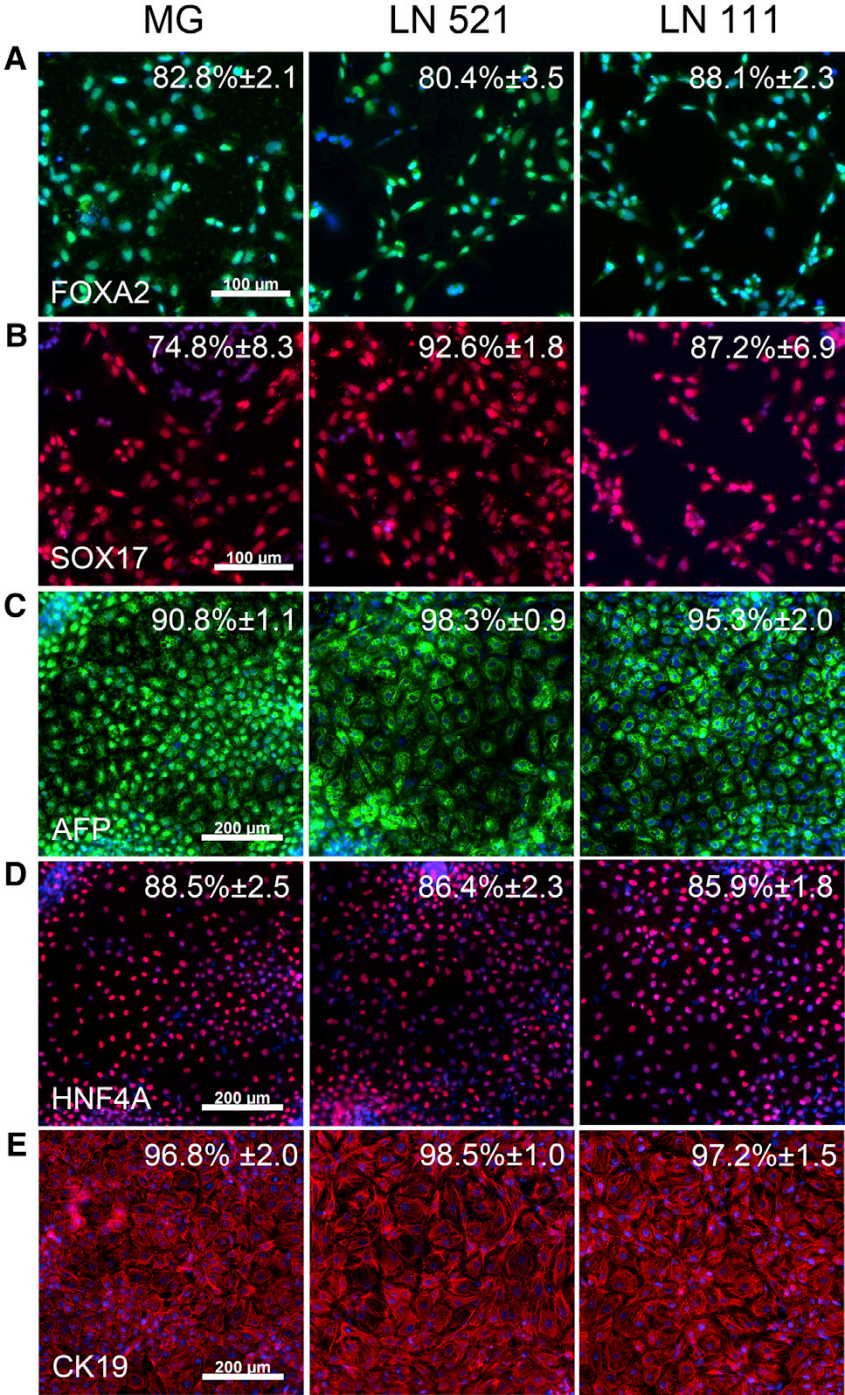
Endoderm and Hepatic Specification (A and B) FOXA2 (A) and SOX17 (B) stained cells on MG, laminin 521, and laminin 111 on day 3 of differentiation. Cells were counterstained with Hoechst 33342. The percentage of positive cells and SD are shown. Images were taken at ×20 magnification. (C–E) AFP (C), HNF4a (D), and CK19 (E) on MG, laminin 521, and laminin 111 on day 9 of differentiation. Cells were counterstained with Hoechst 33342. Data are presented as the mean of three independent experiments ± SD. All images were taken at ×10 magnification.

Twenty-four hours after replating, differentiation was initiated using a serum-free procedure (Szkolnicka et al., 2014a, Cameron et al., 2015). Cell extracts were collected, and mRNA was assessed at the denoted time points (Figure 2; Table S2). All differentiation procedures delivered cell populations that transited from pluripotency, through definitive endoderm, to hepatoblast-like cells, and, subsequently, hepatocytes, as demonstrated by qPCR (Figure 2). The pluripotency-associated markers *OCT4* (Figure 2A) and *NANOG* (Figure 2B) decreased over time on all substrates. The endoderm-associated gene *FOXA2* peaked on all substrates on day 3 and then decreased thereafter (Figure 2C). Expression of *AFP* varied across all substrates (Figure 2D). Cells on Matrigel and laminin 521 followed the same trend in expression, peaking on day 9 and then decreasing on day 18. Conversely, cells on the laminin 111 mix gradually increased in *AFP* expression over time, peaking on day 18. *HNF4A* was detected as early as day 3 on all substrates (Figure 2E). By day 9, differences in expression patterns emerged. On Matrigel and laminin 521, *HNF4A* was highest on day 9 and decreased on day 18, whereas, on the laminin 111 mix, on day 18, *HNF4A* expression was increased. Variation in *HNF4A* gene expression was observed between experimental replicates. However, this did not appear to affect downstream differentiation and was not reflected in HNF4α protein levels (Figures 3 and 6).

Expression of *ALB* was upregulated from day 9 across all substrates (Figure 2F). Levels of *ALB* from days 9–18 saw the highest increase on the laminin 111 mix (∼10,000-fold), significantly higher than on Matrigel (p < 0.001) and laminin 521 (p < 0.001).

### Human Embryonic Stem Cell-Derived Hepatoblast Specification

Although our previous data provided encouragement that stem cell differentiation was proceeding as we would expect, our next objective was to test the efficiency of the process on human recombinant laminin 521 and the laminin 111 mix in comparison with Matrigel (Figure 3). Human embryonic stem cell (ESC)-derived endoderm specification was measured using two commonly used endoderm-associated markers: FoxA2 and Sox17. The majority of cells on Matrigel, laminin 521, and laminin 111 mix were positive for FoxA2, with 82.8% ± 2.1%, 80.4% ± 3.5%, and 88.1% ± 2.3% cells positive, respectively (Figure 3A). Sox17 staining was more varied across the three matrices. It was lowest on Matrigel (74.8% ± 8.3%) and higher on laminin 521 (92.6% ± 1.8%) and the laminin 111 mix (87.2% ± 6.9%) (Figure 3B). As differentiation progressed and hepatic fate was specified (by day 9), cells began expressing high levels of hepatoblast markers. AFP was expressed in the majority of cells on Matrigel (90.8% ± 1.1%), laminin 521 (98.3% ± 0.9%), and the laminin 111 mix (95.3% ± 2.0%) (Figure 3C). The trend was similar for HNF4A, with the majority of cells expressing the transcription factor on Matrigel (88.5% ± 2.5%), laminin 521 (86.4% ± 2.3%), or the laminin 111 mix (85.9% ± 1.8%) (Figure 3D). CK19 expression was also observed in the majority of cells differentiated on Matrigel (96.8% ± 2.0%), laminin 521 (98.5% ± 1.0%), and the laminin 111 mix (97.2% ± 1.5%) (Figure 3E). Hepatoblast specification on all three matrices appeared to be equivalent and highly efficient, and the initial differences in hESC morphology observed on the three matrices did not appear to affect the kinetics or the efficiency of cellular differentiation. However, we did observe an ∼2-fold increase in cell size in HLCs differentiated on the laminin 111 mix and 521 substrates (Figure 3E, CK19).

### Human Embryonic Stem Cell-Derived Hepatocyte Specification and Maturation

**Figure 4:**
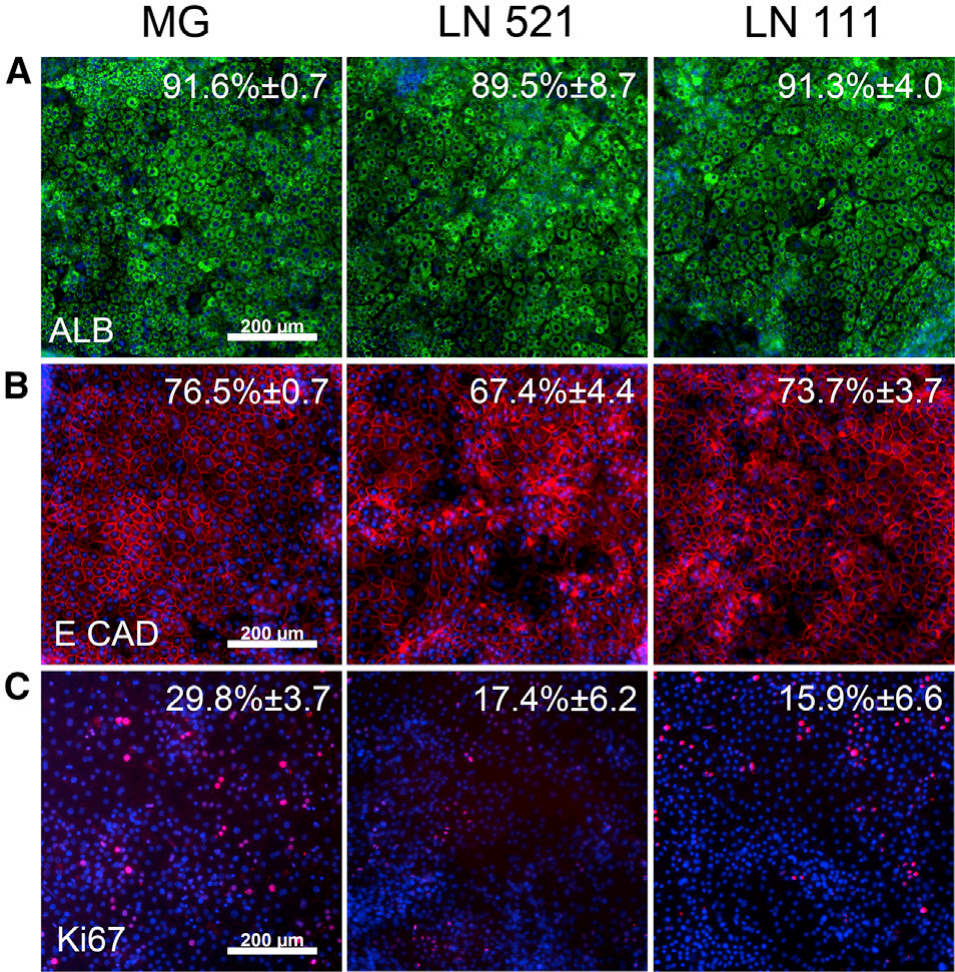
Hepatocyte Maturation (A–C) Albumin (A), E Cadherin (B), and cellular proliferation marker (Ki67, C) staining of cells on MG, laminin 521, and laminin 111 on day 18 of differentiation. Cells were counterstained with Hoechst 33342. Data are presented as the mean of three independent experiments ± SD. All images were taken at ×10 magnification.

Post-hepatoblast specification, cell cultures were differentiated toward hepatocytes, and, on day 18, hepatocyte specification was assessed by immunostaining for albumin and E-cadherin. Notably, similar patterns of protein production in the Matrigel and laminin populations were observed (Figure 4). Albumin staining was detected in HLCs on Matrigel (91.6% ± 0.7%), laminin 521 (89.5% ± 8.7%), and the laminin 111 mix (91.3% ± 4.0%) (Figure 4A). Next, E-cadherin (E CAD) expression was assessed because of its importance in cell-to-cell contact (Treyer and Müsch, 2013) and low-level expression in fetal hepatocytes (Stamatoglou et al., 1992). On Matrigel and the laminin 111 mix, E-cadherin was expressed in 76.5% (± 0.7%) of cells and 73.7% (± 3.7%), whereas HLCs on laminin 521 were 67.4% (± 4.4%) positive (Figure 4B). Although immunostaining studies showed equivalence between the cell populations, differences in cell proliferation were observed on the different substrates. Of note, HLCs derived on Matrigel demonstrated an increase in the cell proliferation marker Ki67, with 29.8% (± 3.7%) of HLCs staining positive. This was in contrast to 17.4% (± 6.2%) of positive HLCs on laminin 521 and 15.9% (± 6.6%) on the laminin 111 mix (Figure 4C).

### Human Embryonic Stem Cell-Derived Hepatocyte and Primary Hepatocyte Function

**Figure 5:**
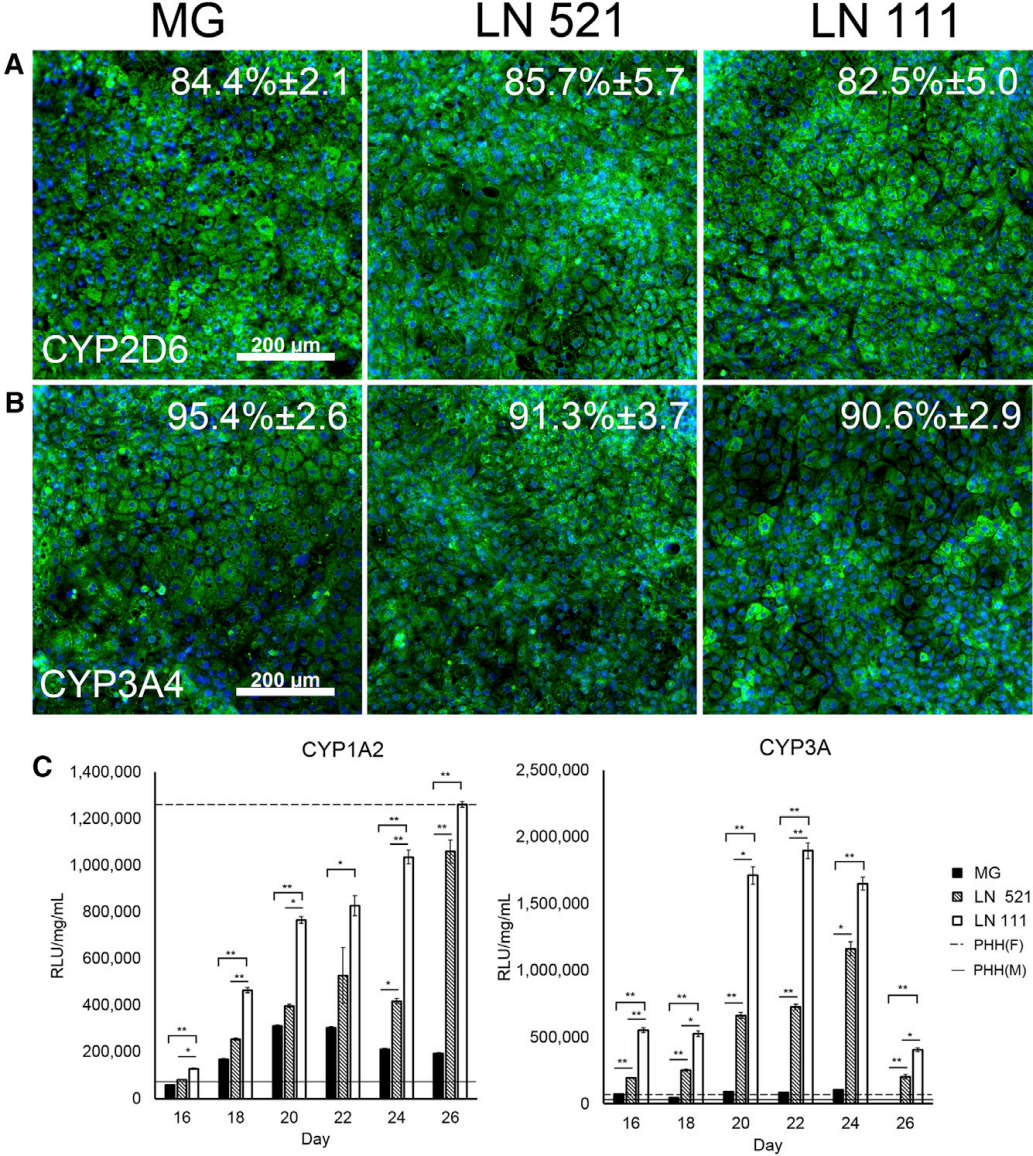
Metabolic Function and Expression (A and B) Expression of CYP2D6 (A) and CYP3A4 (B) on MG, LN521, and LN111 at day 24 of differentiation. Cells were counterstained with Hoechst 33342. Data are presented as the mean of three independent experiments ± SD. All images were taken at ×10 magnification. (C) Cytochrome P450 metabolic activity of CYP1A2 and CYP3A of cells cultured on Matrigel (black columns), laminin 521 (shaded columns), and the laminin 111 mix (white columns). Data are presented as mean of six independent experiments. Error bars represent SEM.^∗^p < 0.05, ^∗∗^p < 0.01; one-way ANOVA with Bonferroni post hoc analysis. The dotted and continuous lines indicates the average CYP activity of primary hepatocytes in culture. PHH(F), primary human hepatocytes, female; PHH(M), primary human hepatocytes, male.

Given that cell division is an important factor in hepatocyte function, we studied hepatocyte metabolic capacity in vitro. Stem cell-derived hepatocytes were examined for cytochrome P450 expression using well characterized antisera. CYP2D6 and CYP3A expression was detected in 84.4% (± 2.1%) and 95.4% (± 2.6%) of HLCs derived on Matrigel (Figures 5A and 5B). On laminin 521, 85.7% (± 5.7%) of cells expressed CYP2D6 and 91.3% (± 3.7%) expressed CYP3A (Figures 5A and 5B). On the laminin 111 mix, 82.5% (± 5.0%) cells were positive for CYP2D6, and 90.6% (± 2.9%) of cells were positive for CYP3A (Figures 5A and 5B). Although protein expression appeared to be equivalent, stem cell-derived hepatocyte CYP P450 function varied (Figure 5C). On the laminin 111 mix, CYP1A2 activity increased over time and was significantly higher than on Matrigel at all time points. On laminin 521, CYP1A2 was elevated at all time points and was significantly greater than on Matrigel cultures on days 24 and 26 of differentiation. CYP3A function was increased up to 25-fold on the laminin 111 mix (Figure 5C). HLCs on both laminins exhibited significantly increased metabolic function relative to cells on Matrigel at all time points. To corroborate these data, differentiation experiments were carried out in another hESC line, Manchester 12, available at GMP-grade (Figure S4). The results obtained demonstrate that HLCs derived from Manchester 12 (Man12) on both laminins displayed significantly superior CYP3A and CYP1A2 function in comparison with Matrigel cultures (Figure S4).

Following this, stem cell-derived hepatocyte function was compared with cryopreserved adult hepatocytes. Primary hepatocyte function varied between male and female samples (continuous versus dotted line in Figure 5C). CYP1A2 function was comparable between primary hepatocytes and HLCs generated on the laminin 111 mix (Figure 5C; Figure S3C), whereas CYP3A function was significantly superior to primary hepatocytes in HLCs differentiated on both laminins (Figure 5C; Figure S3C). Although both H9- and Man12-derived hepatocytes displayed improved function on laminin 521 and the laminin 111 mix, H9-derived hepatocytes were closer in terms of metabolic activity to cryopreserved human hepatocytes (Figure 5; Figure S4). Despite these changes in metabolic capacity, we did not detect any significant differences in albumin or α fetoprotein secretion by ELISA (Figure S2). This was in contrast to another hESC line, Manchester 11 (Man11). Man11-derived hepatocytes did not demonstrate improved cytochrome P450 activities on laminin 521 and the 111 mix. However, they did demonstrate organized features and significantly reduced AFP secretion when compared with Matrigel, demonstrating a reduction of fetal-like behavior. In support of this, Man11-derived populations also displayed significantly increased albumin secretion on laminin 521 in comparison with Matrigel and the laminin 111 mix (Figure S5).

### Human Embryonic Stem Cell-Derived Hepatocyte Organization

**Figure 6:**
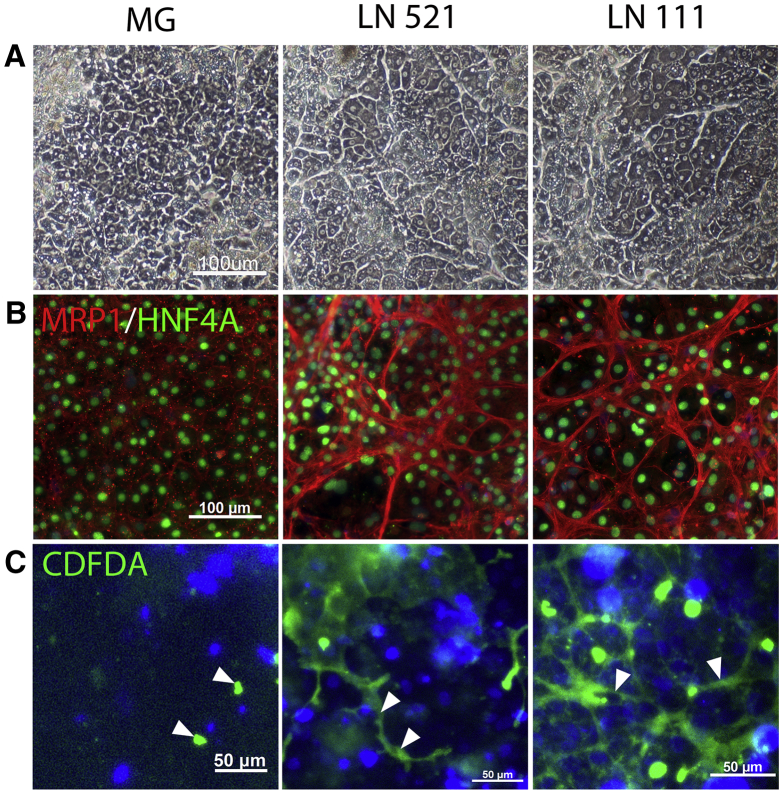
Functional Organization of Hepatocyte-like Cells (A) Phase contrast images of cells on day 24 of culture on MG, LN521, and LN111. (B) Co-immunostaining of MRP-1 (red) and of HNF4a (green). (C) CDFDA staining shows functional bile canaliculi on laminins, whereas, on Matrigel, only diffuse staining is seen. Cells were counterstained with Hoechst 33342, and all images were taken at ×10 magnification. The data presented are representative of three independent experiments.

HLCs derived on laminin 521 and the laminin 111 mix displayed a more primary hepatocyte-like appearance with very pronounced nuclei that were often bi-nucleate (Figure 6A). Phase contrast images also indicated that hepatocytes were arranged in organized structures within the culture dish. Around these hepatic clusters, an important basal membrane marker was detected, multidrug resistance-associated protein (MRP-1). Only hepatocytes differentiated on laminins 521 and the laminin 111 mix exhibited networks of organized hepatocytes expressing MRP1 in vitro. This was in stark contrast to cells on Matrigel, which displayed more individual and punctate staining (Figure 6B). To determine whether cell organization improved canalicular function, we examined biliary efflux using 5(6)-carboxy-2′,7′-dichlorofluorescein diacetate (CDFDA). Notably, cell organization was paralleled by more active biliary efflux in cells differentiated on laminin 521 and the laminin 111 mix versus Matrigel (Figure 6C).

### Genome-wide Analysis

**Figure 7:**
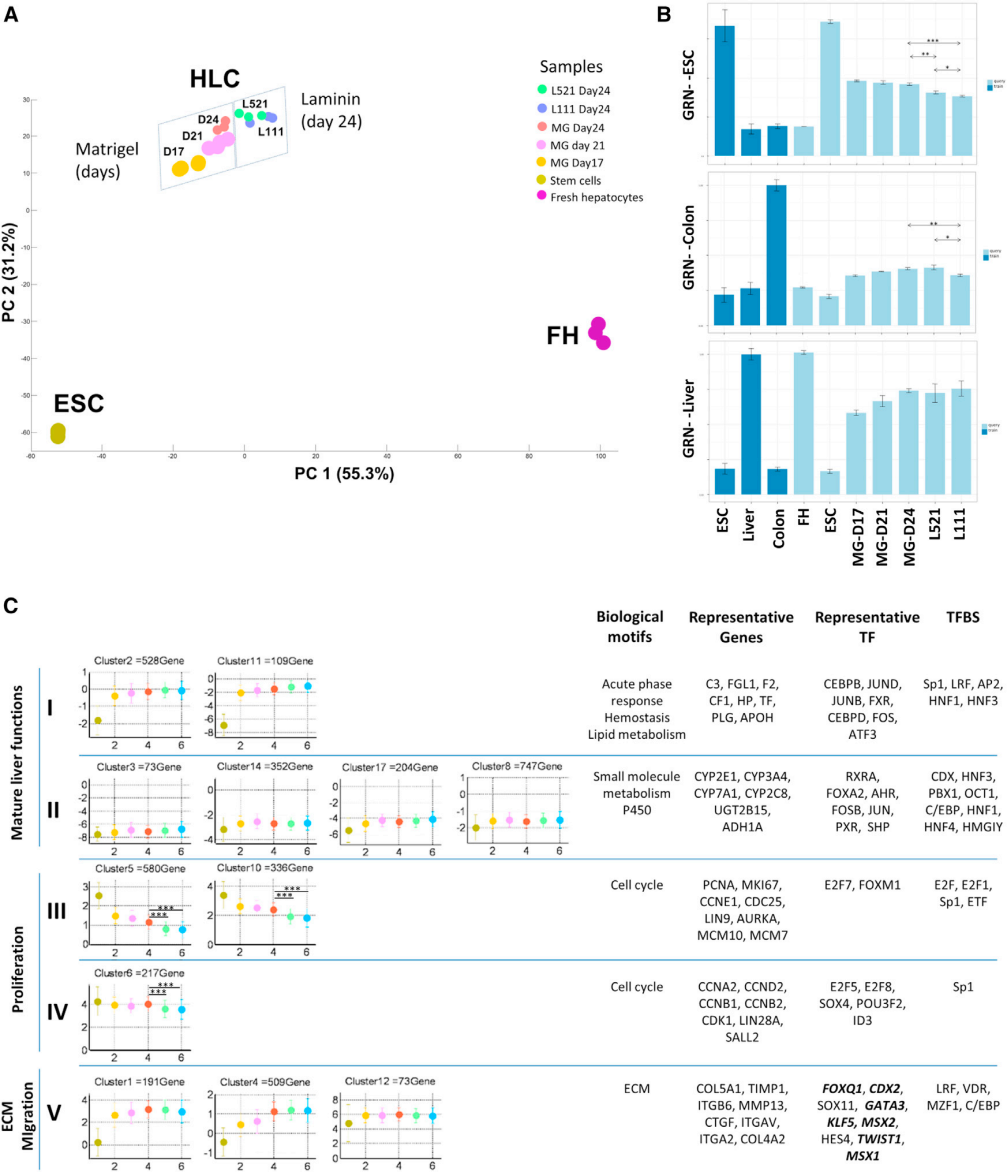
Gene Array Analysis Identifies Genome-Wide Effects of the Laminin Matrix in HLC Differentiation (A) Principal component analysis of the 1,000 genes with highest variance in hESCs, FHs, and HLCs differentiated on MG for 17, 21, and 24 days or L111 or L521 for 24 days. The results shown represent three biological replicates. The graph shows the two largest principal components that constitute 86.5% of the variance. (B) GRN status obtained from the gene expression profile in FHs, ESCs and HLCs on the different matrices. The training score for ESCs, colon, and liver are shown in dark blue and represent the maximal score for each cell/tissue. The score for the queried samples (in light blue) is calculated in relation to the maximal cell/tissue specific score, as described in Cahan et al. (2014). Significantly different scores were observed for laminin 521 and laminin 111 versus Matrigel (day 24) on ESCs and Colon GRNs (^∗∗∗^p < 0.005, ^∗∗^p < 0.05, ^∗^0.5, t test). (C) Clustering of genes with similar expression patterns in HLCs generated three superclusters representing the motifs “mature liver functions,” “proliferation,” and “ECM/migration” (see Supplemental Information for details). Shown are the KEGG and gene ontology (GO) motifs overrepresented in each gene cluster. Representative genes for each cluster and motif are also indicated. Genes in cluster V were expressed at lower levels on HLCs in laminin 111 compared with Matrigel. See Table S3 for a full list of gene and motif enrichment analyses in each gene cluster. Data are presented as the mean of three independent experiments. significant differences in mean gene expression levels (log2 scale) were observed between Matrigel (day 24), laminin 521, and laminin 111 in clusters 5, 10, and 6, which correspond to the supercluster proliferation (t test, p < 0.005).

The experiments presented so far demonstrated an improvement in stem cell differentiation to hepatocytes on laminins. To understand which gene regulatory networks underpinned this, we performed an extensive and unbiased bioinformatics analysis (Figure 7). For this purpose, hESCs were differentiated on Matrigel (Godoy et al., 2015), laminin 521, and the laminin 111 mix. The standard differentiation protocol was applied, and whole-genome expression profiles of three independent experiments were analyzed. Data were compared with a previous study (Godoy et al., 2015) that used freshly isolated primary human hepatocytes (FHs), hESC cells, and Matrigel-differentiated HLCs (day 17 and 21). These data were compared with stem cell-derived hepatocytes derived on Matrigel, laminin 521, and the laminin 111 mix on day 24 of the differentiation process (day 24, L521 and L111, respectively).

The overview by principal component analysis illustrates that laminin-directed differentiation shifted HLCs toward FHs (Figure 7A). The number of differentially expressed genes in this study (556 between Matrigel and laminin 521 and 664 between Matrigel and the laminin 111 mix, false discovery rate [FDR] adjusted) could be considered major. To further characterize this difference, we employed CellNet software (Cahan et al., 2014; Figure 7B). Consistent with previous studies (Godoy et al., 2015, Morris et al., 2014), CellNet identified repression of stem cell gene regulatory network scores (GRN-ESC) and increased liver and colon GRNs in stem cell-derived hepatocytes (Figure 7B). Importantly, CellNet demonstrated a significant decrease of the hESC- and colon-associated GRNs in laminin- versus Matrigel-differentiated samples (Figure 7B; Figure S6). Of the two laminins, the laminin 111 mix showed a significantly stronger effect than pure laminin 521 (Figure 7B). To obtain further insights, we used a recently published fuzzy clustering technique (Godoy et al., 2015). In the proliferation- and cell migration-associated clusters, laminins led to a significantly stronger suppression than Matrigel cultures (clusters III–V; Figure 7C). These results were confirmed by qPCR with a reduction in E2F7, AURKA, SOX11, and TFAP2A on laminin versus Matrigel (Figure S6). A reduction in stem cell (SALL2 and LIN28A) and fibroblast (FOXF2) gene expression was also detected by qPCR. In summary, the main advantage of laminin over Matrigel was a more efficient suppression of inappropriate GRNs controlling stem cell biology, colon specification, fibroblast specification, and cell proliferation and migration (Figure 7; Figure S6).

Given the important role of integrins as receptors for laminins and their ability to bind EGF, an important liver mitogen, we examined integrin expression and phosphatidylinositol 3-kinase (PI3K)-Akt and Jak-STAT signaling in hepatocytes replated on each ECM. In these analyses, we focused on the genes in the Kyoto Encyclopedia of Genes and Genomes (KEGG) categories “PI3K-AKT Signaling Pathway,” “JAK-STAT Signaling Pathway,” and “ECM-Receptor Interaction.” KEGG pathway analysis identified a significant overrepresentation of the aforementioned KEGG gene clusters in all HLCs (Table S3; Figure S7). From the bioinformatics analyses, we could not identify clear changes in GRNs that may be the drivers of enhanced hepatocyte differentiation on recombinant laminins (Figure 7B; Figure S7A), and this requires more detailed investigation. Likewise, the ECMs used in our study had subtle effects on integrin expression. ITGB2 and ITGAL gene expression was downregulated in comparison with freshly isolated hepatocytes, whereas other integrins were upregulated, including ITGAV, ITGA3, and ITGB6 (Figure S7C). Notably, we did detect that integrins ITGA7 and ITGA6 were differentially regulated on laminin versus Matrigel, and this will be the focus of a future investigation (Figure S7C).

## Discussion

The extracellular matrix can have profound effects on cells, modulating many biological processes, including cell attachment, migration, proliferation, differentiation repair, and development (Martinez-Hernandez and Amenta, 1993a, Martinez-Hernandez and Amenta, 1993b, Martinez-Hernandez and Amenta, 1995). By mimicking key elements of the liver cell niche using two laminin isoforms, we dramatically improved hepatocyte differentiation, significantly enhancing cell organization and function. Although similar numbers of HLCs were produced on the three matrices, distinct differences in HLC phenotype, organization, and function were observed. Notably, CYP1A2 and 3A function were equivalent or superior to primary human hepatocytes when stem cell-derived hepatocytes were cultured on laminin 521 and the laminin 111 mix, and this remained stable for several days in culture.

In addition to metabolic function, stem cell-derived hepatocytes displayed more organized structures on the laminin substrates. The differences in cell organization between laminin and Matrigel cultures could have been underpinned by the upregulation of key proliferation and motility genes on Matrigel. With the exception of E2F7, which was reduced significantly on the laminin 111 mix only, AURKA, SOX11, and TFAP2A were reduced on both laminins. E2F7 is an important regulator of hepatocyte proliferation and human telomerase gene expression (Sirma et al., 2011) and represents a key target in the quest for a mature and stably differentiated hepatocyte in vitro. Similarly, the suppression of AURKA, SOX11, and TFAP2A, known to play key roles in cell proliferation, hyperplasia, and suppression of terminal differentiation, were also suppressed on both laminin substrates (Gadi et al., 2013, Holl et al., 2011, Lee et al., 2013), representing significant progress. Enhanced hepatocyte organization on both laminins was evidenced by MRP1 staining and resulted in improved canalicular excretion of CDFDA, suggesting a mature feature stem cell-derived hepatocytes in vitro (Karpen and Suchy, 2001).

The analysis of liver gene expression and function suggested an improvement of the hepatocyte phenotype on laminins. We were keen to understand what underpinned this and employed gene microarrays. Using principal component analysis, our first goal was to understand whether the different matrices cause dramatic or only minor changes in overall gene expression (Godoy et al., 2009, Godoy et al., 2010). The number of differentially expressed genes in this study could be considered major, with the laminin 111 mix and laminin 521 imparting three specific features on HLCs compared with Matrigel. First, expression of pluripotency-associated transcripts was more effectively suppressed by laminins. Second, the laminin 111 mix more efficiently suppressed proliferation and migration-associated gene expression. Third, induction of unwanted colon- and fibroblast-associated gene expression, which is an inherent side effect of the currently used hepatocyte differentiation protocols (Godoy et al., 2015), was ameliorated by the laminin 111 mix.

Alongside improved function and a decrease in unwanted gene expression, these laminins also provide a xeno-free alternative to Matrigel. As such, serum-free directed hepatocytes, in combination with laminin 521 or the laminin 111 mix, can now be described as “defined.” To test whether our procedure was compatible with hESC lines available at clinical grade, we employed two Manchester hESC lines (Man11 and Man12). All three lines differentiated efficiently, with the majority of cells expressing albumin and CYP3A. The same level of cellular organization was also demonstrated across the GMP lines, with the HLCs on laminin 521 and the laminin 111 mix displaying networks of MRP1 staining. Notably, the H9-derived hepatocytes displayed a closer metabolic profile to primary hepatocytes than Man11- and -12-derived hepatocytes. However, both laminin 521 and the laminin 111 mix did improve the Man11 and Man12 HLC phenotype, demonstrating significant progress.

In conclusion, the efficient specification and superior performance of stem cell-derived hepatocytes on laminin substrates and their compatibility with GMP hESC lines demonstrate an important advance. We believe that our approach to hepatocyte differentiation will allow the cost-effective production of GMP-grade hepatocytes at scale that could ultimately be used in the clinic should they deemed to be fit for the purpose.

## Experimental Procedures

### Cell Culture

H9, Man11, and Man12 (hESCs) were cultured as described previously (Szkolnicka et al., 2013) and maintained in a humidified 37°C, 5% CO_2_ incubator. Human ESCs were plated onto pre-coated Matrigel, 5 μg/cm^2^ laminin 521, or 5 μg/cm^2^ laminin 111 mix (a blend of laminins 521 and 111 at a 1:3 ratio). This ratio was suggested as optimal by the laminin supplier (Biolamina). Differentiation was initiated at 40% confluence by replacing serum-free medium mTESR1 (STEMCELL Technologies) with endoderm differentiation medium: RPMI 1640 containing 1× B27 (Life Technologies), 100 ng/mL Activin A (PeproTech), and 50 ng/mL Wnt3a (R&D Systems). The medium was changed every 24 hr for 72 hr. On day 4, endoderm differentiation medium was replaced with hepatoblast differentiation medium, and this was renewed every second day for a further 5 days. The medium consisted of knockout (KO)-DMEM (Life Technologies), Serum replacement (Life Technologies), 0.5% Glutamax (Life Technologies), 1% non-essential amino acids (Life Technologies), 0.2% β-mercaptoethanol (Life Technologies), and 1% DMSO (Sigma). On day 9, differentiating cells were cultured in the hepatocyte maturation medium HepatoZYME (Life Technologies) containing 1% Glutamax (Life Technologies), supplemented with 10 ng/ml hepatocyte growth factor (PeproTech) and 20 ng/ml oncostatin m (PeproTech) as described previously (Rodin et al., 2010, Szkolnicka et al., 2014b).

### Primary Human Hepatocyte Culture

Cryoplateable human hepatocytes (Life Technologies) were plated and maintained according to the vendor’s instructions. Briefly, cryoplateable hepatocytes were resuscitated in thawing medium (CM3000) and plated onto Matrigel or laminin pre-coated 96-well plates. Cells attached to all matrices efficiently and were maintained in a humidified 37°C, 5% CO_2_ incubator. 24 hr after plating, the medium was changed to incubation medium (CM4000). 48 hr after replating, hepatocyte metabolic activity was measured using CYP3A4 and CYP1A2 pGlo technology (Promega) as described previously (Szkolnicka et al., 2014b).

### Albumin and α Fetoprotein ELISA

hESC-derived and cryoplateable hepatocyte αfetoprotein and albumin production was quantified using commercially available ELISA kits (Alpha Diagnostic International). The different media were collected at the denoted time points during hESC differentiation (days 20–26). Primary hepatocyte medium was harvested 24 hr after plating onto Matrigel- or laminin-coated surfaces. Samples were run in triplicate and measured on a FLUOStar Omega multi-mode microplate reader (BMG Labtech). Protein production was expressed as nanogram or microgram of protein per milliliter of medium per milligram of protein (bicinchoninic acid [BCA] assay, Pierce).

### RNA Isolation and qPCR

Total RNA was isolated from cells using Trizol reagent and purified in accordance with the manufacturer’s instructions (Life Technologies). RNA quantification and quality were assessed using a Nanodrop system. The Superscript III reverse transcription kit (Life Technologies) was employed to prepare the cDNA. qPCR was performed with TaqMan Fast Advance Mastermix and the appropriate primer pair (Applied Biosystems) and analyzed using a Roche LightCycler 480 real-time PCR system. Gene expression was normalized to glyceraldehyde 3-phosphate dehydrogenase (GAPDH) and expressed as relative expression over the control sample (hESC on day 0 of differentiation). qPCRs were performed in triplicate. Data analysis was performed using Roche LightCycler 480 software (version 1.5). Levels of significance were measured by Student’s t test and defined as p < 0.05.

### Immunofluorescence

Throughout the differentiation process, cell cultures were fixed in 100% ice-cold methanol at −20°C for 30 min. Subsequently, fixed cells were washed twice with PBS at room temperature. Cell monolayers were blocked with 0.1% PBS-Tween containing 10% BSA for 1 hr, and subsequently the monolayers were incubated with primary antibodies diluted in PBS-0.1% Tween/1% BSA at 4°C overnight. The following day, the primary antibody was removed, and the fixed monolayers were washed three times with PBS-0.1% Tween/1% BSA. Following this, the cells were incubated with the appropriate secondary antibody diluted in PBS/0.1% Tween/1% BSA for 1 hr at room temperature and washed three times with PBS. Cultures were then mounted with PermaFluor aqueous mounting medium (Thermo Scientific) and counterstained with NucBlue Hoechst 33342 (Sigma-Aldrich). The cells were imaged with an Axio Observer Z1 microscope with LD PlanNeoFluar objective lenses (Carl Zeiss). This microscope was coupled to a Zeiss AxioCamMR3 camera used for image acquisition. The images were processed through Zeiss Axiovision SE 64 Rel 4.8, with Zeiss Axiovision version 4.9.1.0 used to analyze the images. The percentage of positive cells and SD was estimated from at least five random fields of view.

### Cytochrome P450 Assays

CYP3A and CYP1A2 activity was measured from days 16–26 using pGlo technology (Promega) and carried out according to the manufacturer’s instructions for CYP450 activity estimation. CYP activity was expressed as relative light units (RLUs) per milliliter of medium per milligram of protein (BCA assay, Pierce). Levels of significance were measured by Student’s t test. The experiments with hESC-derived hepatocytes are representative of six biological replicates, whereas the primary human hepatocyte experiments are representative of three biological replicates.

### Functional Polarization Assay

hESC-derived hepatocytes were incubated with 2 μM of CDFDA for 30 min. Cultures were then washed with ice-cold phosphate-buffered saline containing calcium and magnesium. After washing, stem cell-derived hepatocytes were counterstained with DAPI and collected for imaging. CDFDA efflux from canalicular like structures was examined microscopically.

### Gene Microarray and Bioinformatics Studies

Gene microarrays and bioinformatics studies were compared and performed as described previously (Godoy et al., 2015). The extracts used in these studies pertain to day 24 stem cell-derived HLCs on Matrigel, LN521, and the LN111 mix.

## Author Contributions

K.C. designed, performed, and analyzed the experiments and contributed to the writing of the paper. R.T. performed and analyzed the experiments. Y.W., B.L.V., and D.S. optimized previous and validated new procedures presented in this manuscript. G.C. and W.S.H. performed and analyzed microarray and qPCR data. P.G and J.G.H. analyzed microarray and qPCR data and contributed to the writing of the manuscript. M.J.L. carried out experimental statistical analysis. N.B. and S.J.K. derived and provided the GMP-grade cell lines Man11 and Man12. S.J.F provided funding, analyzed experiments, and contributed to the writing of the paper. D.C.H led the conception, design, and analysis of the experiments; wrote the manuscript; and provided funding.

## Accession Numbers

The accession number for the microarray data reported in this paper is EMBL-EBI: E-MTAB-3994.

## Supplemental Information

Document S1. Figures S1–S7 and Tables S1 and S2

Table S3. Selected Gene Clusters, Related to Figure 7

Document S2. Article plus Supplemental Information

## References

[bib1] Basma H., Soto-Gutiérrez A., Yannam G.R., Liu L., Ito R., Yamamoto T., Ellis E., Carson S.D., Sato S., Chen Y. (2009). Differentiation and transplantation of human embryonic stem cell-derived hepatocytes. Gastroenterology.

[bib2] Cahan P., Li H., Morris S.A., Lummertz da Rocha E., Daley G.Q., Collins J.J. (2014). CellNet: network biology applied to stem cell engineering. Cell.

[bib3] Cai J., Zhao Y., Liu Y., Ye F., Song Z., Qin H., Meng S., Chen Y., Zhou R., Song X. (2007). Directed differentiation of human embryonic stem cells into functional hepatic cells. Hepatology.

[bib4] Cameron K., Villarin B.L., Szkolnicka D., Hay D.C., Vinken M., Rogiers V. (2015). Serum-free directed differentiation of human embryonic stem cells to hepatocytes. Protocols in In Vitro Hepatocyte Research.

[bib5] Carlsson R., Engvall E., Freeman A., Ruoslahti E. (1981). Laminin and fibronectin in cell adhesion: enhanced adhesion of cells from regenerating liver to laminin. Proc. Natl. Acad. Sci. USA.

[bib6] Carpentier A., Tesfaye A., Chu V., Nimgaonkar I., Zhang F., Lee S.B., Thorgeirsson S.S., Feinstone S.M., Liang T.J. (2014). Engrafted human stem cell-derived hepatocytes establish an infectious HCV murine model. J. Clin. Invest..

[bib7] Domogatskaya A., Rodin S., Tryggvason K. (2012). Functional diversity of laminins. Annu. Rev. Cell Dev. Biol..

[bib8] Duan Y., Catana A., Meng Y., Yamamoto N., He S., Gupta S., Gambhir S.S., Zern M.A. (2007). Differentiation and enrichment of hepatocyte-like cells from human embryonic stem cells in vitro and in vivo. Stem Cells.

[bib9] Forbes S.J., Gupta S., Dhawan A. (2015). Cell therapy for liver disease: From liver transplantation to cell factory. J. Hepatol..

[bib10] Gadi J., Jung S.H., Lee M.J., Jami A., Ruthala K., Kim K.M., Cho N.H., Jung H.S., Kim C.H., Lim S.K. (2013). The transcription factor protein Sox11 enhances early osteoblast differentiation by facilitating proliferation and the survival of mesenchymal and osteoblast progenitors. J. Biol. Chem..

[bib11] Godoy P., Hengstler J.G., Ilkavets I., Meyer C., Bachmann A., Müller A., Tuschl G., Mueller S.O., Dooley S. (2009). Extracellular matrix modulates sensitivity of hepatocytes to fibroblastoid dedifferentiation and transforming growth factor beta-induced apoptosis. Hepatology.

[bib12] Godoy P., Lakkapamu S., Schug M., Bauer A., Stewart J.D., Bedawi E., Hammad S., Amin J., Marchan R., Schormann W. (2010). Dexamethasone-dependent versus -independent markers of epithelial to mesenchymal transition in primary hepatocytes. Biol. Chem..

[bib13] Godoy P., Schmidt-Heck W., Natarajan K., Lucendo-Villarin B., Szkolnicka D., Asplund A., Björquist P., Widera A., Stöber R., Campos G. (2015). Gene networks and transcription factor motifs defining the differentiation of stem cells into hepatocyte-like cells. J. Hepatol..

[bib14] Hay D.C., Zhao D., Ross A., Mandalam R., Lebkowski J., Cui W. (2007). Direct differentiation of human embryonic stem cells to hepatocyte-like cells exhibiting functional activities. Cloning Stem Cells.

[bib15] Hay D.C., Fletcher J., Payne C., Terrace J.D., Gallagher R.C., Snoeys J., Black J.R., Wojtacha D., Samuel K., Hannoun Z. (2008). Highly efficient differentiation of hESCs to functional hepatic endoderm requires ActivinA and Wnt3a signaling. Proc. Natl. Acad. Sci. USA.

[bib16] Hay D.C., Pernagallo S., Diaz-Mochon J.J., Medine C.N., Greenhough S., Hannoun Z., Schrader J., Black J.R., Fletcher J., Dalgetty D. (2011). Unbiased screening of polymer libraries to define novel substrates for functional hepatocytes with inducible drug metabolism. Stem Cell Res. (Amst.).

[bib17] Holl D., Kuckenberg P., Woynecki T., Egert A., Becker A., Huss S., Stabenow D., Zimmer A., Knolle P., Tolba R. (2011). Transgenic overexpression of Tcfap2c/AP-2gamma results in liver failure and intestinal dysplasia. PLoS ONE.

[bib18] Holmgren G., Sjögren A.K., Barragan I., Sabirsh A., Sartipy P., Synnergren J., Björquist P., Ingelman-Sundberg M., Andersson T.B., Edsbagge J. (2014). Long-term chronic toxicity testing using human pluripotent stem cell-derived hepatocytes. Drug Metab. Dispos..

[bib19] Jitraruch S., Dhawan A., Hughes R.D., Filippi C., Soong D., Philippeos C., Lehec S.C., Heaton N.D., Longhi M.S., Mitry R.R. (2014). Alginate microencapsulated hepatocytes optimised for transplantation in acute liver failure. PLoS ONE.

[bib20] Karpen S.J., Suchy F.J., Suchy F.J., Sokol R.J., Balistreri W.F. (2001). Structural and functional development of the liver. Liver Disease in Children.

[bib21] Lavon N., Yanuka O., Benvenisty N. (2004). Differentiation and isolation of hepatic-like cells from human embryonic stem cells. Differentiation.

[bib22] Lee S.Y., Lee G.R., Woo D.H., Park N.H., Cha H.J., Moon Y.H., Han I.S. (2013). Depletion of Aurora A leads to upregulation of FoxO1 to induce cell cycle arrest in hepatocellular carcinoma cells. Cell Cycle.

[bib23] Lorenzini S., Bird T.G., Boulter L., Bellamy C., Samuel K., Aucott R., Clayton E., Andreone P., Bernardi M., Golding M. (2010). Characterisation of a stereotypical cellular and extracellular adult liver progenitor cell niche in rodents and diseased human liver. Gut.

[bib24] Martinez-Hernandez A., Amenta P.S. (1993). The hepatic extracellular matrix. I. Components and distribution in normal liver. Virchows Arch. A Pathol. Anat. Histopathol..

[bib25] Martinez-Hernandez A., Amenta P.S. (1993). The hepatic extracellular matrix. II. Ontogenesis, regeneration and cirrhosis. Virchows Arch. A Pathol. Anat. Histopathol..

[bib26] Martinez-Hernandez A., Amenta P.S. (1995). The extracellular matrix in hepatic regeneration. FASEB J..

[bib27] Medine C.N., Lucendo-Villarin B., Storck C., Wang F., Szkolnicka D., Khan F., Pernagallo S., Black J.R., Marriage H.M., Ross J.A. (2013). Developing high-fidelity hepatotoxicity models from pluripotent stem cells. Stem Cells Transl. Med..

[bib28] Miner J.H., Li C., Mudd J.L., Go G., Sutherland A.E. (2004). Compositional and structural requirements for laminin and basement membranes during mouse embryo implantation and gastrulation. Development.

[bib29] Morris S.A., Cahan P., Li H., Zhao A.M., San Roman A.K., Shivdasani R.A., Collins J.J., Daley G.Q. (2014). Dissecting engineered cell types and enhancing cell fate conversion via CellNet. Cell.

[bib30] Ng S., Schwartz R.E., March S., Galstian A., Gural N., Shan J., Prabhu M., Mota M.M., Bhatia S.N. (2015). Human iPSC-derived hepatocyte-like cells support Plasmodium liver-stage infection in vitro. Stem Cell Reports.

[bib31] Payne C.M., Samuel K., Pryde A., King J., Brownstein D., Schrader J., Medine C.N., Forbes S.J., Iredale J.P., Newsome P.N., Hay D.C. (2011). Persistence of functional hepatocyte-like cells in immune-compromised mice. Liver Int..

[bib32] Rashid S.T., Corbineau S., Hannan N., Marciniak S.J., Miranda E., Alexander G., Huang-Doran I., Griffin J., Ahrlund-Richter L., Skepper J. (2010). Modeling inherited metabolic disorders of the liver using human induced pluripotent stem cells. J. Clin. Invest..

[bib33] Rodin S., Domogatskaya A., Ström S., Hansson E.M., Chien K.R., Inzunza J., Hovatta O., Tryggvason K. (2010). Long-term self-renewal of human pluripotent stem cells on human recombinant laminin-511. Nat. Biotechnol..

[bib34] Roelandt P., Obeid S., Paeshuyse J., Vanhove J., Van Lommel A., Nahmias Y., Nevens F., Neyts J., Verfaillie C.M. (2012). Human pluripotent stem cell-derived hepatocytes support complete replication of hepatitis C virus. J. Hepatol..

[bib35] Schwartz R.E., Fleming H.E., Khetani S.R., Bhatia S.N. (2014). Pluripotent stem cell-derived hepatocyte-like cells. Biotechnol. Adv..

[bib36] Si-Tayeb K., Noto F.K., Nagaoka M., Li J., Battle M.A., Duris C., North P.E., Dalton S., Duncan S.A. (2010). Highly efficient generation of human hepatocyte-like cells from induced pluripotent stem cells. Hepatology.

[bib37] Siller R., Greenhough S., Naumovska E., Sullivan G.J. (2015). Small-molecule-driven hepatocyte differentiation of human pluripotent stem cells. Stem Cell Reports.

[bib38] Sirma H., Kumar M., Meena J.K., Witt B., Weise J.M., Lechel A., Ande S., Sakk V., Guguen-Guillouzo C., Zender L. (2011). The promoter of human telomerase reverse transcriptase is activated during liver regeneration and hepatocyte proliferation. Gastroenterology.

[bib39] Stamatoglou S.C., Enrich C., Manson M.M., Hughes R.C. (1992). Temporal changes in the expression and distribution of adhesion molecules during liver development and regeneration. J. Cell Biol..

[bib40] Sullivan G.J., Hay D.C., Park I.H., Fletcher J., Hannoun Z., Payne C.M., Dalgetty D., Black J.R., Ross J.A., Samuel K. (2010). Generation of functional human hepatic endoderm from human induced pluripotent stem cells. Hepatology.

[bib41] Sun P., Zhou X., Farnworth S.L., Patel A.H., Hay D.C. (2013). Modeling human liver biology using stem cell-derived hepatocytes. Int. J. Mol. Sci..

[bib42] Szkolnicka D., Zhou W., Lucendo-Villarin B., Hay D.C. (2013). Pluripotent stem cell-derived hepatocytes: potential and challenges in pharmacology. Annu. Rev. Pharmacol. Toxicol..

[bib43] Szkolnicka D., Farnworth S.L., Lucendo-Villarin B., Hay D.C. (2014). Deriving functional hepatocytes from pluripotent stem cells. Curr. Protoc. Stem Cell Biol..

[bib44] Szkolnicka D., Farnworth S.L., Lucendo-Villarin B., Storck C., Zhou W., Iredale J.P., Flint O., Hay D.C. (2014). Accurate prediction of drug-induced liver injury using stem cell-derived populations. Stem Cells Transl. Med..

[bib45] Takayama K., Nagamoto Y., Mimura N., Tashiro K., Sakurai F., Tachibana M., Hayakawa T., Kawabata K., Mizuguchi H. (2013). Long-term self-renewal of human ES/iPS-derived hepatoblast-like cells on human laminin 111-coated dishes. Stem Cell Reports.

[bib46] Takayama K., Kawabata K., Nagamoto Y., Kishimoto K., Tashiro K., Sakurai F., Tachibana M., Kanda K., Hayakawa T., Furue M.K., Mizuguchi H. (2013). 3D spheroid culture of hESC/hiPSC-derived hepatocyte-like cells for drug toxicity testing. Biomaterials.

[bib47] Tanimizu N., Kikkawa Y., Mitaka T., Miyajima A. (2012). α1- and α5-containing laminins regulate the development of bile ducts via β1 integrin signals. J. Biol. Chem..

[bib48] Taylor-Weiner H., Schwarzbauer J.E., Engler A.J. (2013). Defined extracellular matrix components are necessary for definitive endoderm induction. Stem Cells.

[bib49] Touboul T., Hannan N.R., Corbineau S., Martinez A., Martinet C., Branchereau S., Mainot S., Strick-Marchand H., Pedersen R., Di Santo J. (2010). Generation of functional hepatocytes from human embryonic stem cells under chemically defined conditions that recapitulate liver development. Hepatology.

[bib50] Treyer A., Müsch A. (2013). Hepatocyte polarity. Compr. Physiol..

[bib51] Villarin B.L., Cameron K., Szkolnicka D., Rashidi H., Bates N., Kimber S.J., Flint O., Forbes S.J., Iredale J.P., Bradley M., Hay D.C. (2015). Polymer Supported Directed Differentiation Reveals a Unique Gene Signature Predicting Stable Hepatocyte Performance. Adv. Healthc. Mater..

[bib52] Ware B.R., Berger D.R., Khetani S.R. (2015). Prediction of drug-induced liver injury in micropatterned co-cultures containing iPSC-derived human hepatocytes. Toxicol. Sci..

[bib53] Wu X., Robotham J.M., Lee E., Dalton S., Kneteman N.M., Gilbert D.M., Tang H. (2012). Productive hepatitis C virus infection of stem cell-derived hepatocytes reveals a critical transition to viral permissiveness during differentiation. PLoS Pathog..

[bib54] Zhou W., Hannoun Z., Jaffray E., Medine C.N., Black J.R., Greenhough S., Zhu L., Ross J.A., Forbes S., Wilmut I. (2012). SUMOylation of HNF4α regulates protein stability and hepatocyte function. J. Cell Sci..

[bib55] Zhou X., Sun P., Lucendo-Villarin B., Angus A.G.N., Szkolnicka D., Cameron K., Farnworth S.L., Patel A.H., Hay D.C. (2014). Modulating innate immunity improves hepatitis C virus infection and replication in stem cell-derived hepatocytes. Stem Cell Reports.

