# Supplementary material for: Recombinant Laminins Drive the Differentiation and Self-Organization of hESC-Derived Hepatocytes
Source: Stem Cell Reports. 2015 Nov 25;5(6):1250–62. doi: 10.1016/j.stemcr.2015.10.016 (PMC4682209; doi:10.1016/j.stemcr.2015.10.016)
Supplement: Document S1. Figures S1–S7 and Tables S1 and S2 [file mmc1.pdf]

Stem Cell Reports, Volume *5*

Supplemental Information

Recombinant Laminins Drive the Differentiation and Self-Organization of hESC-Derived Hepatocytes

Kate Cameron, Rosanne Tan, Wolfgang Schmidt-Heck, Gisela Campos, Marcus J. Lyall, Yu Wang, Baltasar Lucendo-Villarin, Dagmara Szkolnicka, Nicola Bates, Susan J. Kimber, Jan G. Hengstler, Patricio Godoy, Stuart J. Forbes, and David C. Hay

Supplementary data:

Figure S1: Related to figure 1 and figure 2

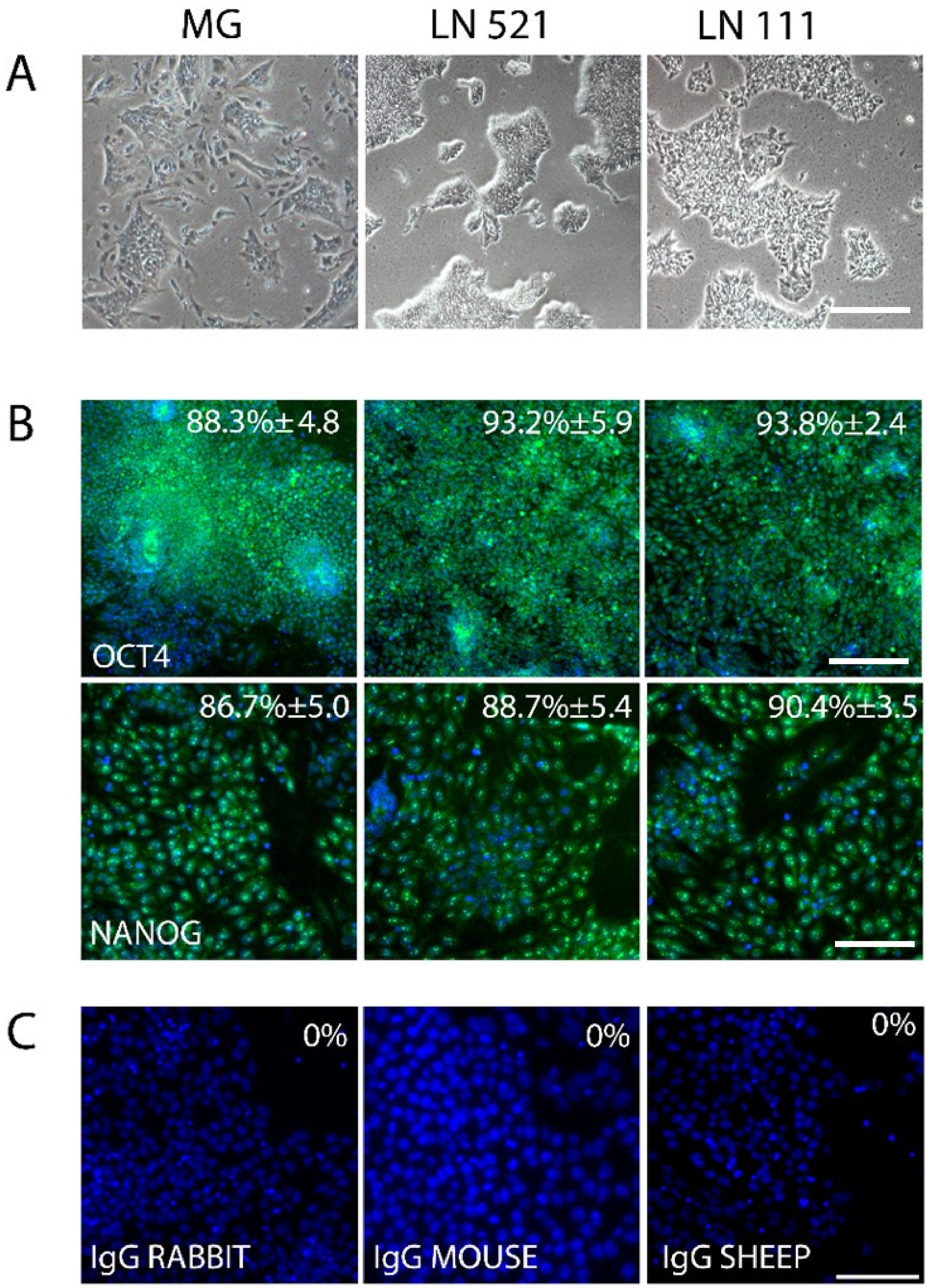

Figure S2: Related to figure 5.

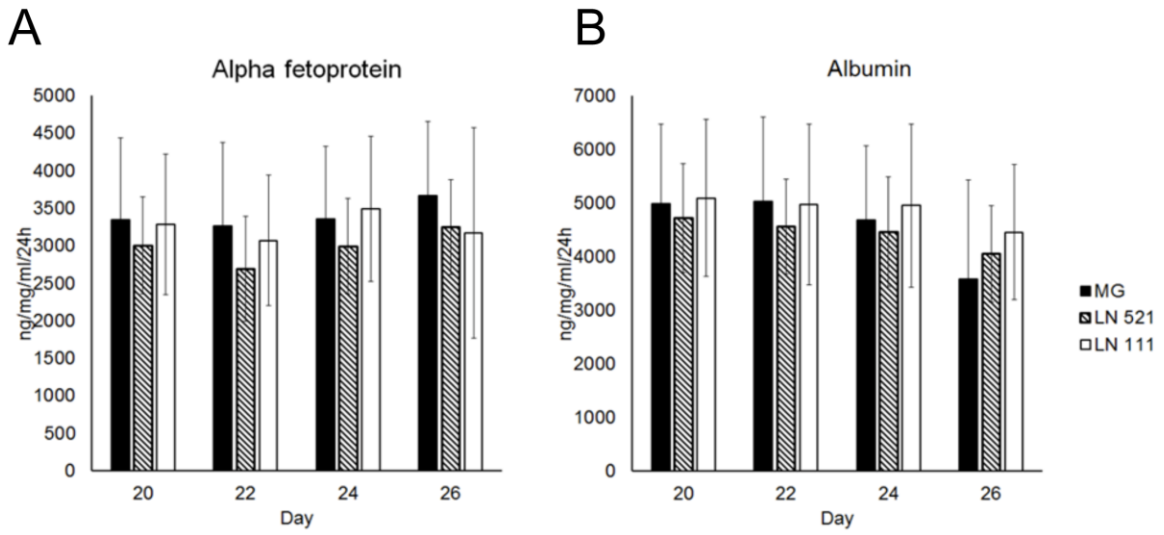

Figure S3: Related to figure 5.

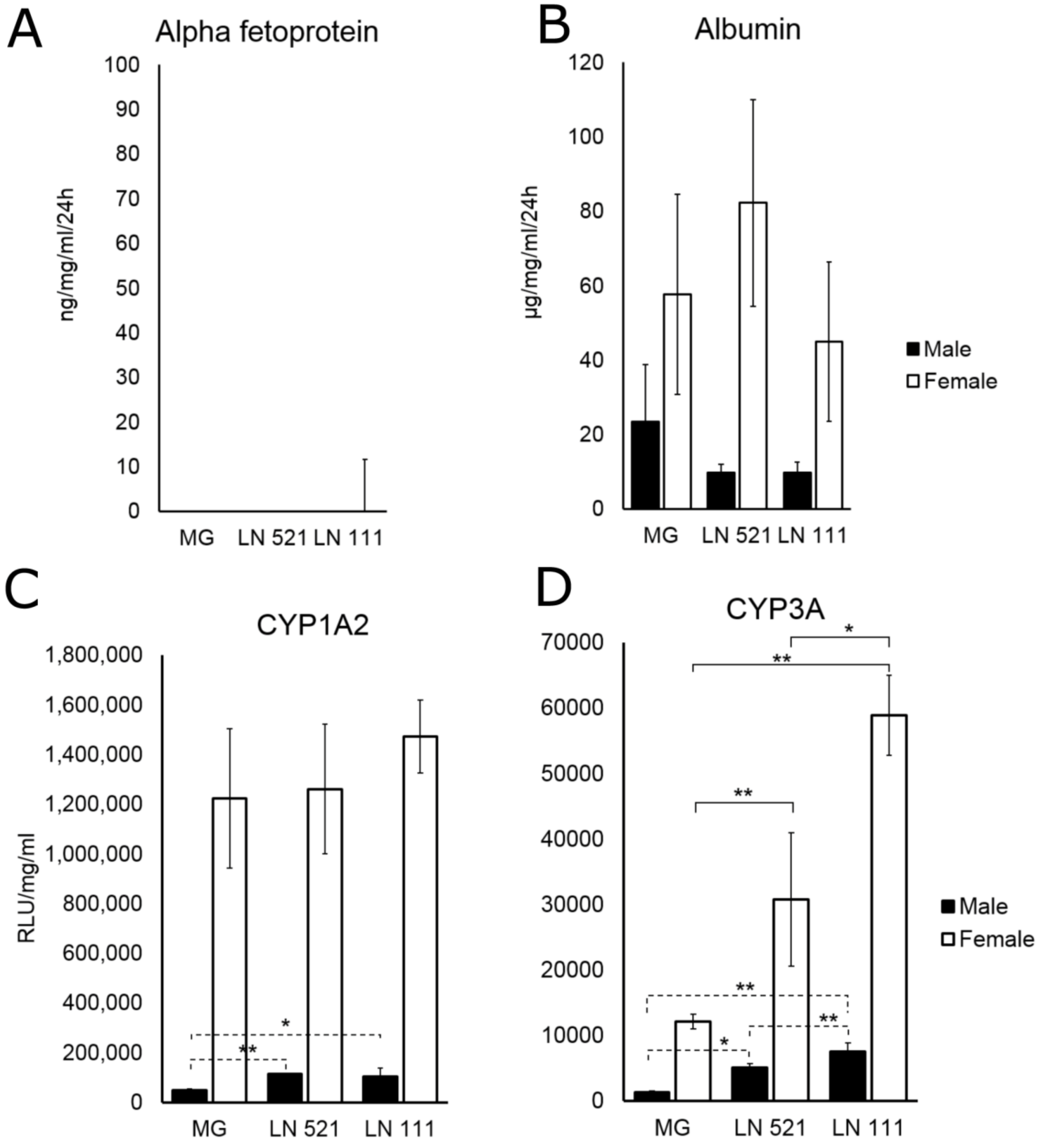

Figure S4: Related to figures 4, 5 and 6.

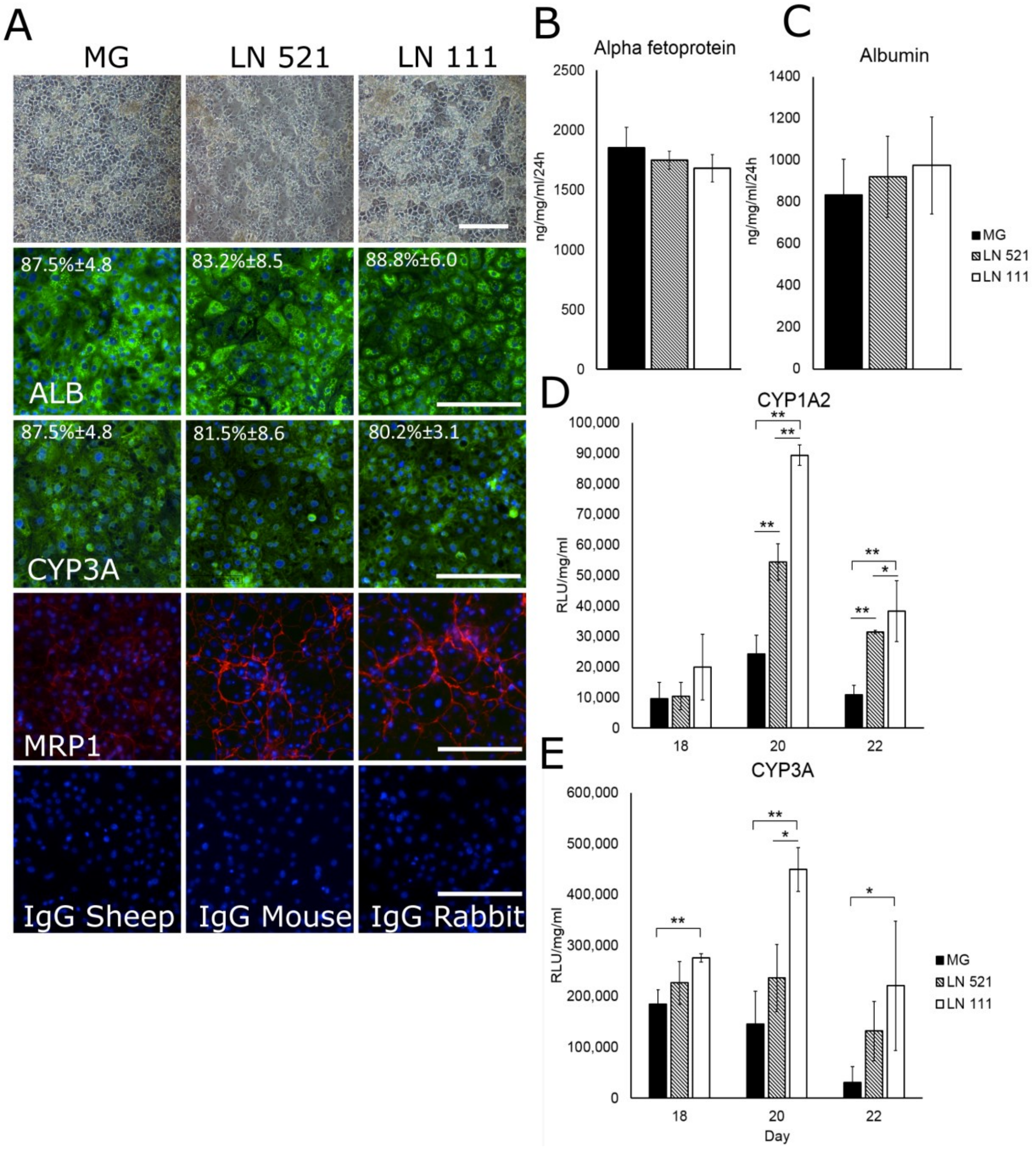

Figure S5: Related to figures 4, 5 and 6.

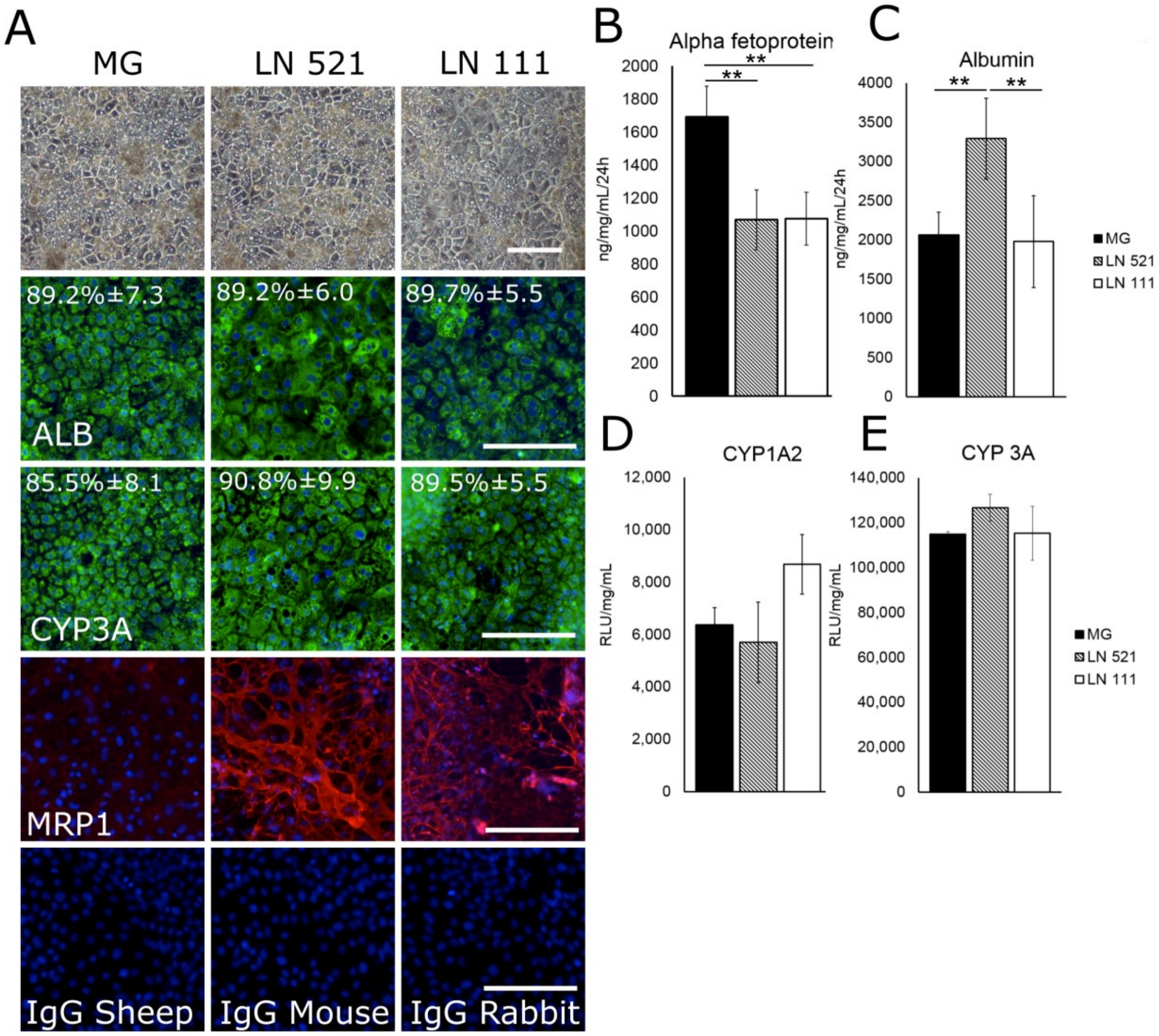

Figure S6: Related to figure 7.

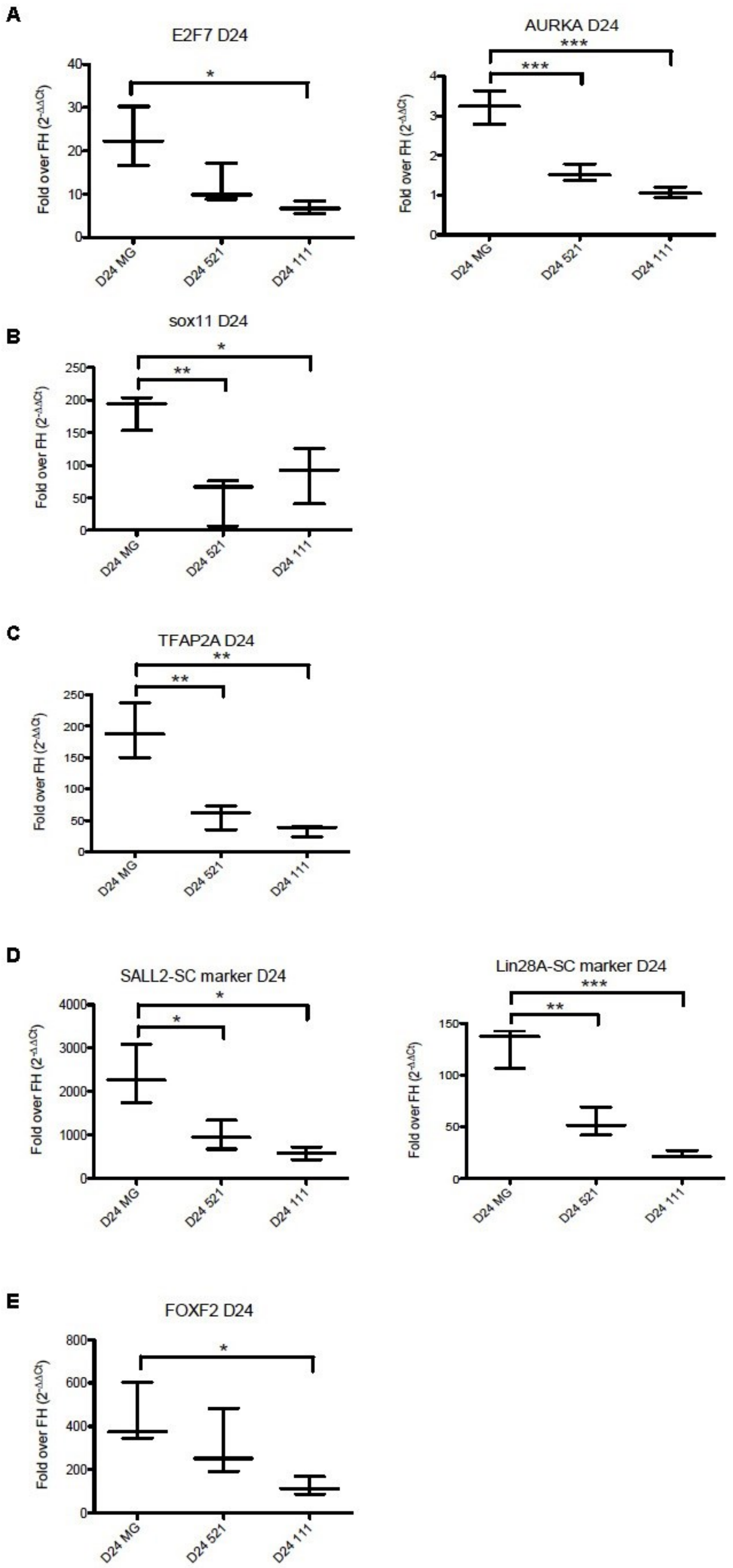

Figure S7: Related to figure 7.

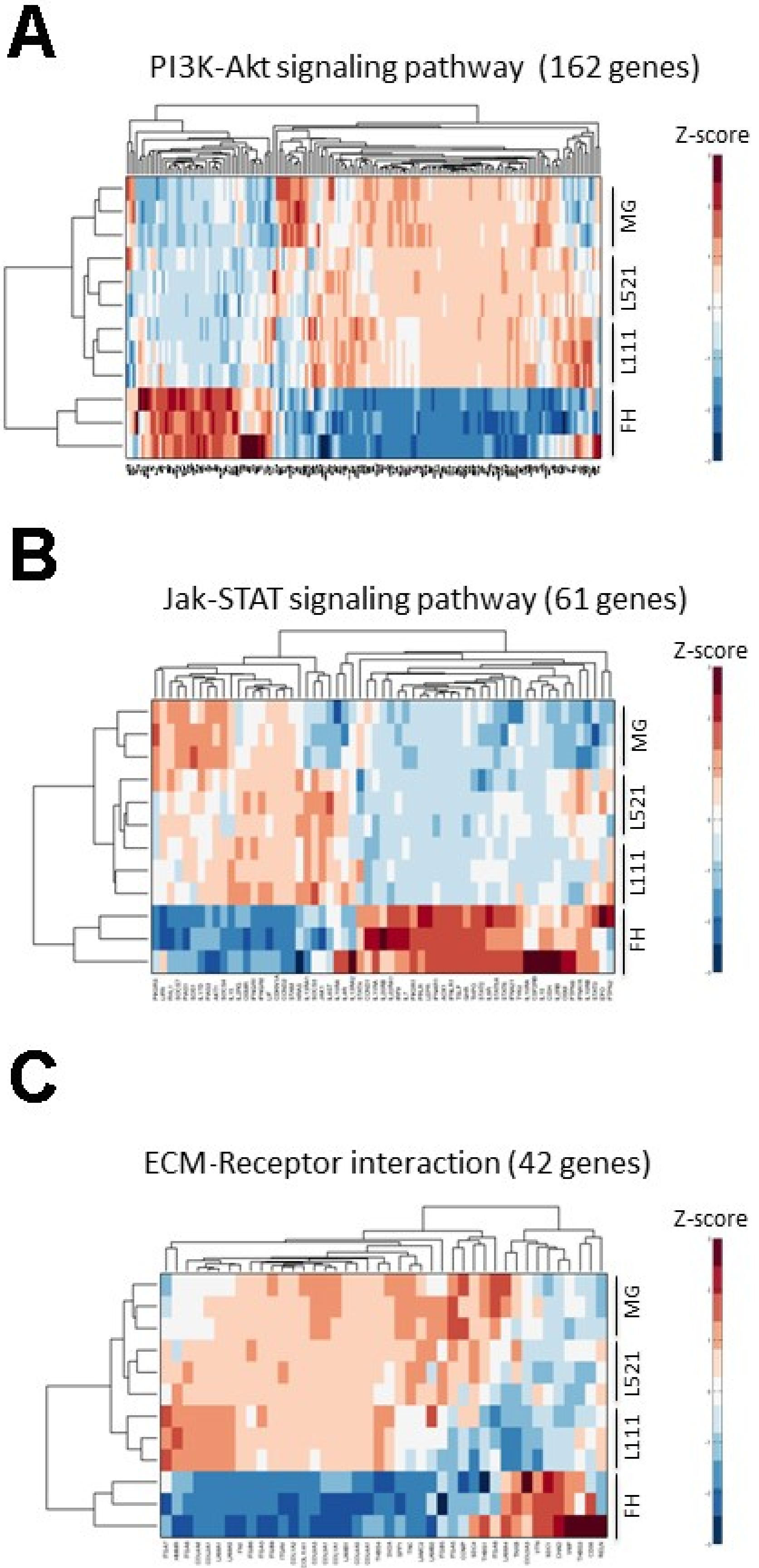

Figure S1: Morphology and Pluripotency marker expression of cells.

(A) Phase contrast images of pluripotent stem cell morphology on Matrigel, laminin 521 and laminin 111 immediately prior to of the onset of differentiation. (B) Immunofluorescent staining of octamer 4 (Oct4) and Nanog protein on Matrigel, laminin 521 and laminin 111 prior to the onset of differentiation (C) Immunoglobulin G control staining for rabbit, mouse and sheep antisera used throughout the manuscript. Data are presented as mean of three independent experiments and the percentage of positive cells and standard deviation is shown. All cells were counterstained with hoescht 33342 and unless stated all images were taken at x10 magnification. The scale bar represents 200µm. Data are presented as the mean of three independent experiments +/- the standard deviation.

Figure S2: Human embryonic stem cell derived hepatocyte protein production.

Hepatocyte protein production of (A) alpha fetoprotein and (B) Albumin of cells cultured on Matrigel (MG), laminin 521 (laminin 521) or laminin 111 mix (laminin 111). Days 20 to 26 during differentiation did not vary significantly between matrices or over time. Data are presented as mean of three independent experiments and error bars represent the standard deviation, there was no statistical significance observed.

Figure S3: Primary human hepatocyte metabolism and protein production.

Primary human hepatocytes (PHHs) were plated onto Matrigel (MG), laminin 521 (LN 521) or laminin 111 mix (LN 111) and hepatocyte protein secretion was analysed by ELISA (A) alpha fetoprotein protein production and (B) Albumin, alpha fetoprotein was not produced at detectable levels and albumin production was enhanced on LN 521 in female PHHs. Protein secretion was not significantly affected across the three substrates. Primary human hepatocytes were also examined for metabolic competence 48 hours post replating, CYP p450 (C) 1A2 and (D) 3A activity was increased on laminins relative to MG in both male (black bars) and female (white bars) PHHs. Data are presented as mean of three independent experiments; error bars represent the standard deviation. *= $P < 0.05$ ** = $P < 0.01$ by students t-test.

Figure S4: Hepatic differentiation and function in GMP grade cell line MAN12.

MAN12 cells were plated onto Matrigel (MG), laminin 521 (LN 521) or laminin 111 mix (LN 111) and morphology assessed by phase microscopy (A). Cells were also examined for hepatic markers (A) Albumin (ALB), cytochrome P450 3A (CYP3A), and multidrug resistance protein 1 (MRP1). All scale bars represent 200µm. Data are presented as mean of three independent experiments and the percentage of positive cells and standard deviation is shown. Hepatocyte protein secretion of (B) alpha fetoprotein and (C) albumin were analysed by ELISA on day 24 with no significant differences observed between matrices. Metabolic function of CYP p450 activity was quantified on days 18, 20 and 24 (D) 1A2 and (E) 3A activity. Data are presented as mean of three independent experiments; error bars represent the standard deviation. * = $P < 0.05$ ** = $P < 0.01$ by students t-test.

Figure S5: Hepatic differentiation and function in GMP grade cell line MAN11.

MAN11 cells were plated onto Matrigel (MG), laminin 521 (LN 521) or laminin 111 mix (LN 111) and morphology assessed by phase microscopy (A). Cells were also examined for hepatic markers (A) Albumin (ALB), cytochrome P450 3A (CYP3A), and multidrug resistance protein 1 (MRP1). All scale bars represent 200μm. Data are presented as mean of three independent experiments and the percentage of positive cells and standard deviation is shown. Hepatocyte protein secretion of (B) alpha fetoprotein and (C) albumin were analysed by ELISA on day 18 with significant differences observed. Metabolic function of CYP p450 activity was quantified on day 18 (D) 1A2 and (E) 3A activity with no significant differences observed between matrices. Data are presented as mean of three independent experiments; error bars represent the standard deviation. * = $P < 0.05$ ** = $P < 0.01$ by students t-test.

Figure S6: qPCR evaluation of stem cell derived hepatocyte gene expression.

Stem cell derived hepatocyte gene expression was studied at Day 24 in the differentiation process on Matrigel (MG), laminin 521 (LN 521) or laminin 111 mix (LN 111). The results from these validation experiments are organised into (A) Custer 3 (E2F7 and AURKA), (B) Cluster 4 (SOX11) (C) Cluster 5 (TFAP2A), (D) Stem Cell (SALL2 LIN28) and (E). Fibroblast gene expression (FOXF2). Data are presented as mean of three independent experiments, and error bars represent the standard deviation. * = $P < 0.05$ ** = $P < 0.01$ one-way ANOVA with Tukey post hoc test.

Figure S7: Cell signalling pathways and integrin expression analysis.

Identification of genes involved in signalling pathways and ECM recognition. (A) PI3K-Akt signaling pathway (B) Jak-STAT signaling pathway and (C) ECM-Receptor interactions. The red colored boxes represent upregulated genes in all HLC. The heat maps show a detailed analysis of gene expression levels in freshly isolated hepatocytes (FH), and in hepatocyte like cells (HLCs) on the laminin 111 mix (L111), laminin 521 (L521) and Matrigel (MG) for the corresponding KEGG gene clusters. The z-score legend indicates the gene expression intensity. Data are presented as mean of three independent experiments.

Table S1: Antibodies for immunofluorescence.

Table S2: Primers for qPCR

Table S3: Selected gene clusters.

Table S1.

Antigen	Manufacturer	Catalogue number	Species	Dilution
FOXA2	Abcam	AB5074	Goat	1:200
SOX17	R&D systems	AF1924	Goat	1:500
AFP	Sigma	A8452	Mouse	1:200
HNF4A	Santa Cruz	SC-8987	Rabbit	1:100
CK19	DAKO	GA61561-2	Mouse	1:50
Albumin (ALB)	Abcam	AB10241	Mouse	1:200
E-cadherin (E CAD)	Abcam	AB1416	Mouse	1:100
Ki67	DAKO	M724029-2	Mouse	1:200
CY3A4	University of Dundee	N/A	Sheep	1:100
CYP2D6	University of Dundee	N/A	Sheep	1:100
MRP1	Abcam	AB24102	Mouse	1:50
Alexa Fluor 488 anti mouse	Life technologies	A-11001	Goat	1:400
Alexafluor 568 anti rabbit	Life technologies	A10042	Donkey	1:400
Alexa Fluor 488 anti sheep	Life technologies	A-11015	Donkey	1:400
Alexafluor 488 anti goat	Life technologies	A-11055	Donkey	1:400

Table S2.

Gene	Source - Primer
**OCT4**	Applied Biosystems - Hs00742896 - s1
**NANOG**	Applied Biosystems - Hs02387400 – g1
**FOXA2**	Applied Biosystems - Hs00232764 – m1
**HNF4A**	Applied Biosystems - Hs01023298 – m1
**AFP**	Applied Biosystems - Hs01040596-m1
**ALB**	Applied Biosystems - Hs00910225 – m1
**B2M**	Applied Biosystems - Hs00984230 – m1

Table S3. Provided in a separate Excel file
